# Optofluidic lens based on electrowetting liquid piston

**DOI:** 10.1038/s41598-019-49560-9

**Published:** 2019-09-10

**Authors:** Lin-Yang Li, Rong-Ying Yuan, Jin-Hui Wang, Lei Li, Qiong-Hua Wang

**Affiliations:** 1 School of Electronics and Information Engineering, Sichuan University, Chengdu, 610065 China; 2 School of Instrumentation and Optoelectronic Engineering, Beihang University, Beijing, 100191 China

**Keywords:** Adaptive optics, Optofluidics

## Abstract

The conventional electrowetting lens usually has one tunable liquid-liquid (L-L) interface. The shape of L-L interface is deformed to get variable focal length due to electrowetting effect. However, contact angle saturation of the L-L interface is an unavoidable problem which prevents focal length from further changing. Here, we demonstrate an optofluidic lens based on electrowetting liquid piston. The proposed lens has two connected chambers, the piston chamber and the lens chamber to form a closed-loop fluidic system. The electrowetting liquid piston can generate clockwise and counter-clockwise liquid flows, which can make the L-L interface convex and concave. To prove the concept, we fabricate an optofluidic device whose shortest negative and positive focal lengths are ~−17.9 mm and ~18 mm with 5 mm aperture, respectively. The proposed optofluidic lens has large tunable focal length range. Widespread application of such an adaptive lens is foreseeable.

## Introduction

Adaptive lenses are considered as an emerging technology due to its unique merits, such as tunable focal length, compact structure, and no mechanical moving parts. Usually it deforms its interface between two phases to change the optical power. In the past decades, many driving mechanisms of the adaptive lenses have been proposed. Among them, electrowetting effect is one of the most mature methods^1–6^. A voltage is used to drive the adaptive lens. Therefore, it not only has low power consumption, but also is easy to integrate into electronic products. However, due to the contact angle saturation, the focal length tuning range of electrowetting lens is usually limited^4^. In recent years, many methods have been proposed to increase the focal length tuning range. For example, The PDMS (Polydimethylsiloxane) membrane lens can obtain a wide range of changes^7–12^. However, when the lens is placed vertically, the PDMS membrane interface may distort due to gravity effect, which reduces the image quality of the lens. In order to increase optical power, an annular folded electrowetting liquid lens is proposed, which reflects incident light three times by the reflective film^6^. However, the light loss is serious due to the circular reflection film. The liquid lens system, which uses two or more interfaces, is also a solution to get high power^13^. But the structure of the lens system is usually bulky. In addition, an adaptive lens based on mechanical-wetting is proposed to increase the tuning range of focal length^14^. It adjusts the volume ratio of the two chambers mechanically, resulting in the change in the shape of the liquid-liquid (L-L) interface at the junction of the two chambers. Obviously, it is not easy to integrate into electronic products, which limits its application in imaging system. Therefore, new methods to increase the focal length tuning range of adaptive lenses are still worth studying.

In this paper, we propose an optofluidic lens based on electrowetting liquid piston. The proposed lens has two connected chambers, the piston chamber and the lens chamber, to form a closed-loop fluidic system. In the piston chamber, conductive liquid is surrounded by the immiscible insulating liquid. The conductive liquid can be moved up and down like a piston due to electrowetting effect, which generates clockwise or inverse hour liquid flows. Thus, the shape of the L-L interface at the annular slice is deformed in the lens chamber. The proposed lens combines the advantages of electrowetting lens and mechanical-wetting lens. Therefore, it can provide a large focal length tuning range with good image quality by voltage. We fabricate a prototype, and the measured focal length tuning range is from ~−17.9 mm to infinity and from infinity to ~18 mm with 5 mm aperture. Driven by the voltage, it can change the focal length towards both positive and negative directions to increase the range of focal length.

## Schematic and Principle

**Figure 1:**
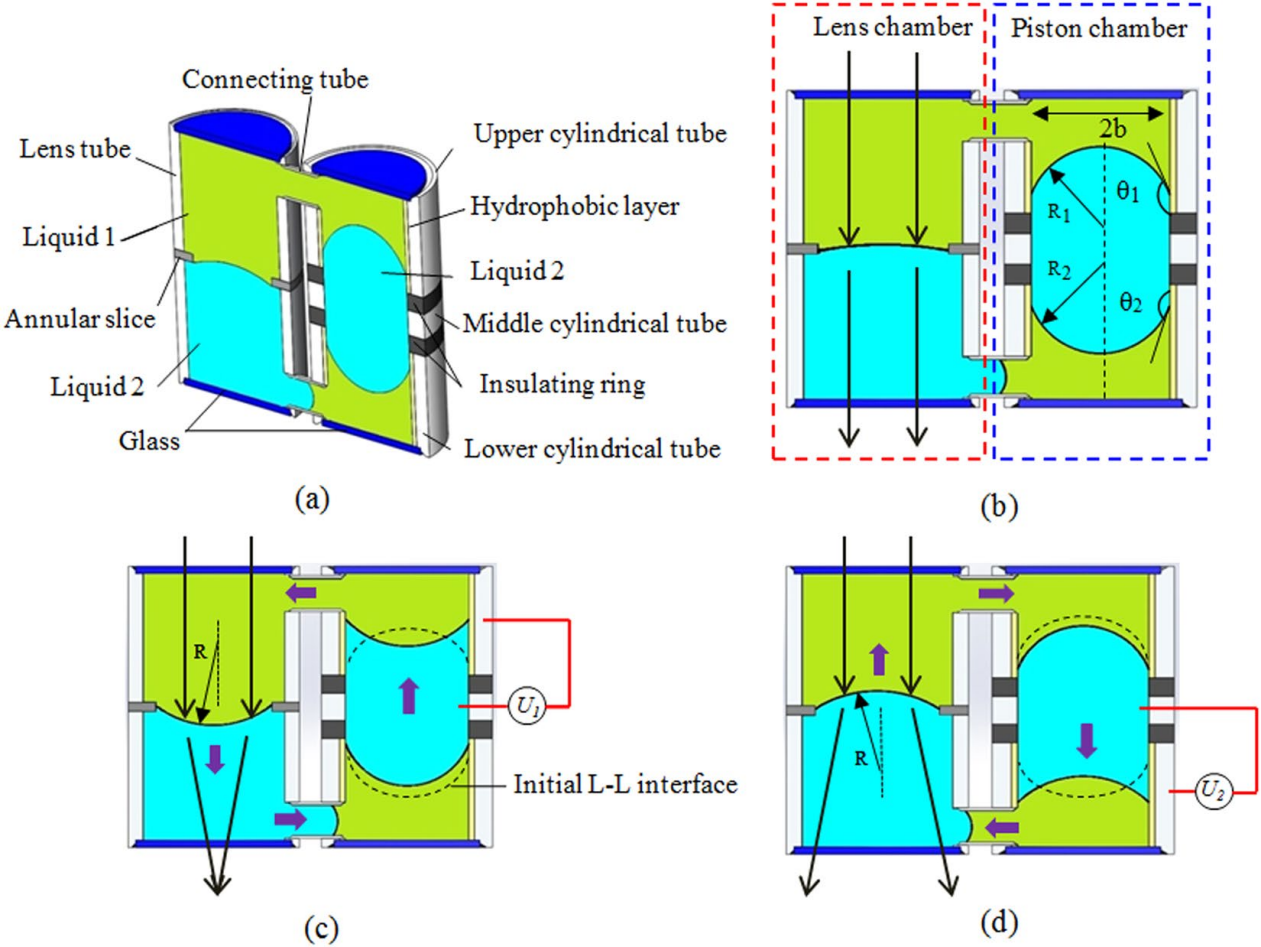
Schematic cross-sectional structure and the principle of changing the focal length: (**a**) Cell structure. (**b**) Initial equilibrium state. (**c**) The equilibrium state when an extra voltage (*U*_1_) is applied to form a positive lens. (**d**) The equilibrium state when an extra voltage (*U*_2_) is applied to form a negative lens.

Figure 1(a) shows a schematic cross section of our proposed optofluidic lens. It consists of a lens chamber and a piston chamber. The two chambers are connected by two tubes to form a closed-loop fluid system. The lens chamber is composed of two cylindrical tubes. In the middle of the lens chamber, there is an annular slice, where the L-L interface is formed. The piston chamber is composed of an upper cylindrical tube, a lower cylindrical tube, two insulating rings and a middle cylindrical tube. The upper cylindrical tube, lower cylindrical tube and middle cylindrical tube serve as three electrodes. In the lens chamber, Liquid 1 and Liquid 2 with the same density are filled to form an L-L interface. In the piston chamber, Liquid 2 is surrounded by Liquid 1 to form an “O” shaped liquid piston. In initial state, the liquid piston is located in the middle part of the piston chamber, as shown in Fig. 1(b). When a voltage is applied to the upper cylindrical tube, the contact angle of the Liquid 2 is changed due to electrowetting effect, which in turn alters the capillary pressure. And the capillary pressure pulls the Liquid 2 upward, which generates inverse hour liquid flow and pushes the Liquid 1 down in the lens chamber. Thus, the L-L interface is deformed at the aperture, as shown in Fig. 1(c). When a voltage is applied to the lower cylindrical tube, the liquid piston moves downward, thus, the generated clockwise liquid flow pushes the Liquid 2 in the lens chamber upward at the aperture, which results in a focal length change as shown in Fig. 1(d). The refractive index of the Liquid 1 is greater than Liquid 2, thus a positive lens or a negative lens can be formed when a voltage is applied to the upper or lower electrodes. As a result, the focal length of the lens varies by a voltage.

The focal length of the proposed optofluidic lens is changed by driving the liquid piston. The liquid piston is driven based on electrowetting effect. According to the Young–Lippmann equation^2^, the relationship of the contact angle *θ* and the applied voltage *U* can be described as follows:

1 $$\cos \,\theta =\frac{{\gamma }_{1}-{\gamma }_{2}}{{\gamma }_{LL}}+\frac{{\varepsilon }_{0}{\varepsilon }_{r}}{2{\gamma }_{LL}d}{U}^{2}$$

where *γ*_*LL*_ is the surface tension of Liquid 1/Liquid 2, *θ* is the contact angle, *γ*_1_ and *γ*_2_ are the interfacial tensions of the hydrophobic layer/Liquid 1 and hydrophobic layer/Liquid 2, respectively. *ε*_0_ is dielectric constant in vacuum, *ε*_*r*_ is the relative dielectric constant of the insulating layer, *d* represents the thickness of the insulating layer. From Eq. 1, we can see that contact angle *θ* decreases as voltage *U* increases.

The lens chamber and the piston chamber are connected by two tubes to form a closed-loop system. In an equilibrium state, there is a balance between the surface tension of the four L-L interfaces, as shown in Fig. 1(b). According to the Young-Laplace equation^15^, the balance can be expressed as:

2 $$\frac{2{\gamma }_{LL}}{R}=\frac{2{\gamma }_{LL}\,\cos \,{\theta }_{1}}{b}-\frac{2{\gamma }_{LL}\,\cos \,{\theta }_{2}}{b}+\Delta p$$

where *R* is the mean curvature radius of the L-L interface at the annular slice. b is the radius of the driving chamber. Δ*p* is the pressure difference near the L-L interface in the connecting tube, and it is a constant. In the initial state, *θ*_1_ is equal to *θ*_2_, and *R* is approximately infinite. Therefore, the value of Δ*p* is small enough to be negligible. Combining the relationship between focal length *f* and curvature radius *R*, the Eqs (1) and (2) we get:

3 $$\frac{1}{f}=\pm \frac{{\varepsilon }_{0}{\varepsilon }_{r}({n}_{1}-{n}_{2})}{2b{\gamma }_{LL}d}{U}^{2}$$

When forming a positive lens, we take a positive sign. When forming a negative lens, we take a negative sign. From Eq. (3), we can see that the focal length *f* can be changed in both positive and negative directions by applying a voltage *U*_1_ on the upper electrode or applying a voltage *U*_2_ on the lower electrode, respectively. Thus the tuning range of the focal length is increased.

### Operating process

**Figure 2:**
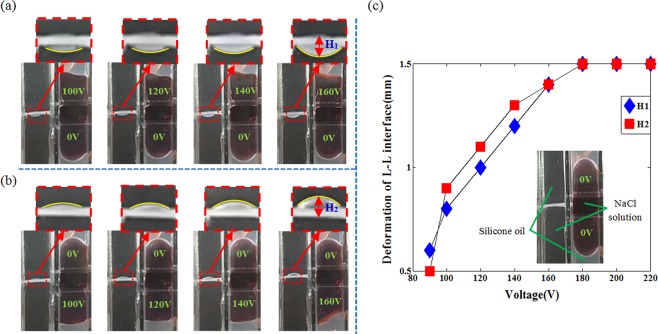
Operating process of the proposed lens. (**a**) The equilibrium state when an extra voltage (*U*_1_) is applied to the upper electrode. (**b**) The equilibrium state when an extra voltage (*U*_2_) is applied to the lower electrode. (**c**) Deformation of L-L interface with different voltages (Video 1).

To visually demonstrate the operating process, we first fabricate the device using transparent materials, as shown in Fig. 2. For the piston chamber, we use four transparent conductive glass sheets (Indium-Tin-Oxide, ITO) to form a cubic chamber, and the conductive area of each piece of glass sheet is engraved into three parts, and two parts are coated with a 3 um Parylene - C + Teflon(AF-1600, Dupont) film as the hydrophobic insulating layer. Then three PMMA (Polymethyl methacrylate) plates and two glass sheets are combined with the piston chamber to form a lens chamber. The annular slice is made of the polytetrafluoroethylene (PTFE) with ~5 mm aperture. Liquid 1 is silicone oil (Phenylmethylsilicone fluid, Dow Corning), and its density is ~1.09 g/cm^3^. Liquid 2 is NaCl solutions, and its density is ~1.09 g/cm^3^. In order to observe the working mechanism of the liquid piston clearly, in the piston chamber, the NaCl solution is dyed brown by edible colorant. A CCD camera which is used to record the deformation of the L-L interface during actuation, is placed on the lateral side of the device. In the initial state, the L-L interface is approximately planar. When the DC voltage (*U*_1_) is applied to the upper electrode in the piston chamber, as shown in Fig. 2(a), the L-L interface is deformed downwards. As the driving voltage increases, the deformation (*H*_1_) of the L-L interface becomes larger. When the voltage increased to 160 V, the deformation (*H*_1_) becomes ~1.4 mm. When further increasing the voltage, the deformation (*H*_1_) remains the same because the contact angle of the Liquid 2 in the piston chamber is saturated, as shown in Fig. 2(c). In the same way, when the DC voltage (*U*_2_) is applied, the L-L interface becomes convex upward to form a negative lens as shown in Fig. 2(b). The largest change in the deformation (*H*_2_) is ~1.5 mm. The variation trend of L-L interface deformation with voltage is similar when applied voltage to the upper or lower electrodes. A dynamic response video of the principle demonstration is also included (Video 1). We also find that, the L-L interface is good in the lens chamber, while the L-L interface in the piston chamber is deviated from our simple model, especially when the voltage is increased to 160 V, as shown in Fig. 2. It mainly results from the fabrication issues. The thickness of the coated hydrophobic insulating layer in different area is not identical. Thus, the L-L interface is distorted in the piston chamber. From above analysis, we can conclude that the shape of the L-L interface can be deformed by driving the liquid piston.

### Fabrication and assembly

**Figure 3:**
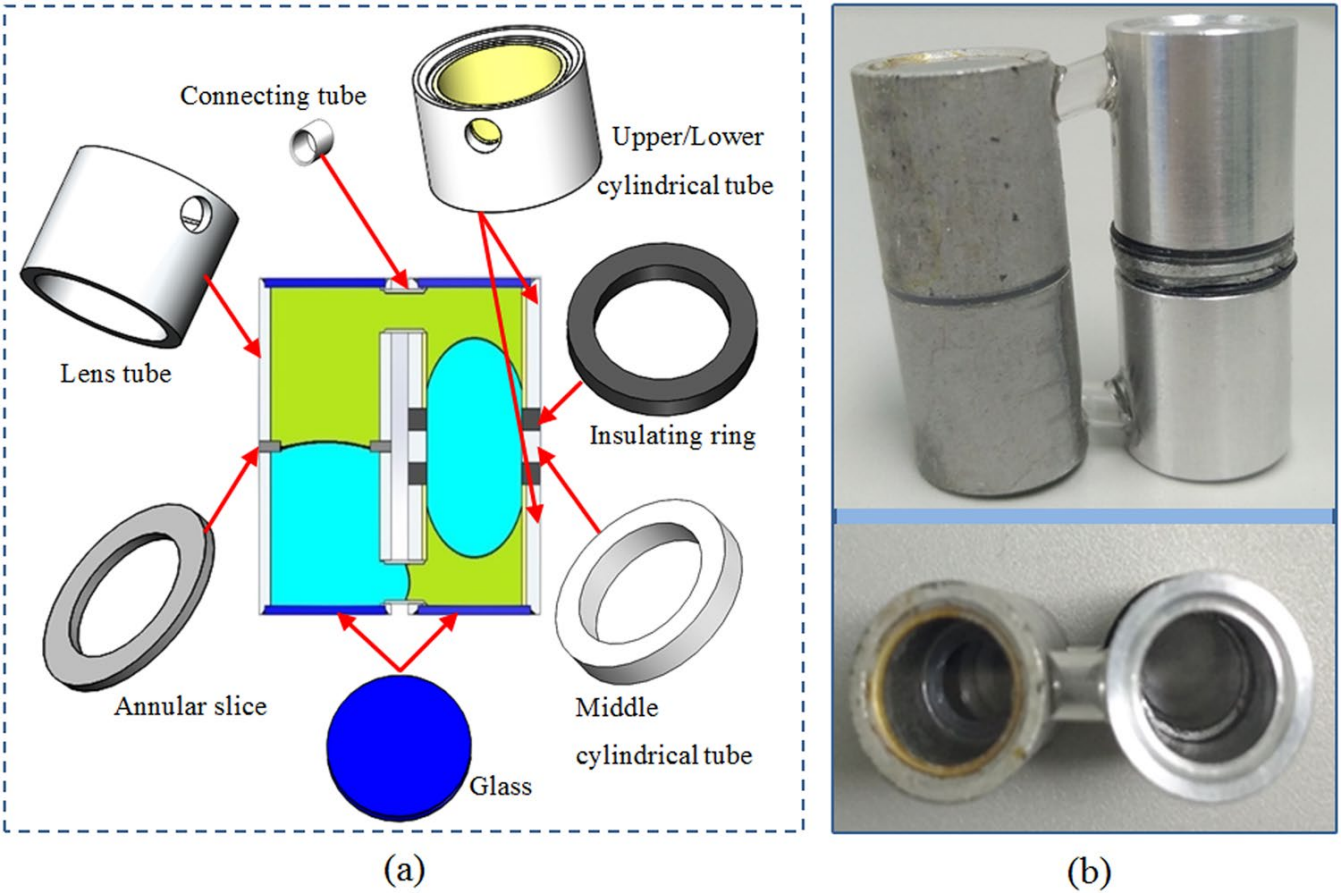
Fabricated prototype of optofluidic lens based on electrowetting piston. (**a**) All the elements of the device. (**b**) Assembled prototype.

To evaluate the performance more accurately, we fabricate a prototype of the proposed lens by precision machining, as shown in Fig. 3(a). Upper/Lower cylindrical tube and middle cylindrical tube are made of aluminum which serves as the electrode of the device. The wall of the cylindrical aluminum tube is coated with Parylene-C and Teflon (AF-1600, Dupont) as the hydrophobic and insulating layer. The thickness of the coated Parylene-C + Teflon is ~3 μm. The material of insulation ring is nylon and the annular slice is made of PTFE. The thickness of the annular slice is 0.5 mm, and the diameter of the circular hole is 5 mm. The material of the top and bottom glass sheets is BK7 in glass data of SHOTT^16^. And all the elements are stuck together to form the prototype by using UV 331 glue. The diameter of the glass sheets are ~8 mm and the height of the entire lens is ~32 mm, as shown in Fig. 3(b). The physical properties of the NaCl solutions and silicone oil are shown in Table 1.

**Table 1:** The physical parameters of the NaCl solutions and silicone oil.

Property	Refractive index	Abbe number	Density (g/cm^3^)
NaCl solutions	1.35	59	1.09
Silicone oil	1.50	38	1.09

### Experiment

**Figure 4:**
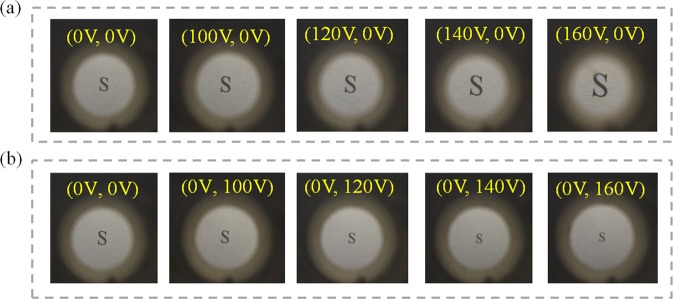
The focal length of the optofluidic lens changes at different voltages. (**a**) Changes in focal length when different voltages are applied to the upper electrode. (**b**) Changes in focal length when different voltages are applied to the lower electrode (Video 2).

We fabricate a prototype to test the imaging quality and zooming ability. The target “S” is placed under the proposed lens. The object distance is 25 mm. A CCD camera is placed behind the proposed lens to collect the image of the target. In the initial state, no voltage is applied, the L-L interface is approximately horizontal, and the size of the image is very close to the object. When a voltage *U*_1_ is applied on the upper electrode, a positive lens is formed. When the voltage increases, the image is magnified, as shown in Fig. 4(a). When the voltage (*U*_1_) reaches ~160 V, the magnification of the image reaches the maximum. When the voltage is removed, the L-L interface returns to the initial state. The negative lens is formed when we apply a voltage *U*_2_ on the lower electrode and the image becomes smaller as the voltage increases, as shown in Fig. 4(b). The image does not change until the voltage increases to ~160 V. Compared the image in Fig. 4(a) with the image in Fig. 4(b), the largest magnification is ~2.5×. In the process of applying voltage, no obvious aberrations were observed. When each voltage is applied to the liquid lens, the CCD camera needs to be refocused to get a clear image. Video 2 shows the image captured by the proposed lens when applying 160 V voltage. The defocusing phenomenon is just the demonstration of the strong zoom ability of our liquid lens. Nevertheless, the proposed lens has only one L-L interface and the spherical aberration is unavoidable. Therefore, the aberration can be corrected by combining several lenses. From the experiment, we conclude that the proposed lens can achieve a large positive and negative turning range of focal length by voltages.

**Figure 5:**
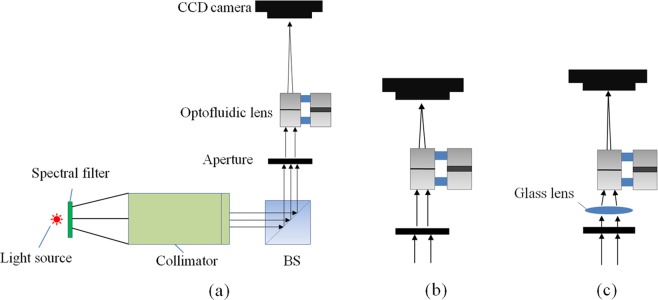
Focal length measurement of the optofluidic lens. (**a**) Schematic diagram of experimental device. (b) An experimental device for measuring positive lenses. (**c**) An experimental device for measuring negative lenses.

To measure the focal length of the proposed lens, we set up a system to test focal length. The system consists of a collimator, a beam splitter (BS), a glass lens, a CCD camera and the proposed lens. The setup is shown in Fig. 5(a). For positive state, a collimated beam passes the BS and the proposed lens, as shown in Fig. 5(b). We adjust the back focal distance to find the focal point. Then the distance between the center of the lens and the image plane is the focal length. For negative lens, as shown in Fig. 5(c), a glass lens is used to collect the incident light. The distance between the glass lens and the proposed lens is 34 mm. The focal length of the glass lens is 20 mm. We first measure the focal length of the lens system. Then, the focal length of the negative lens is calculated as follows^17^:

4 $$f^{\prime} =({f^{\prime} }_{1}-d)\times {f^{\prime} }_{0}/({f^{\prime} }_{1}-{f^{\prime} }_{0})$$

*f*′ is the focal length of the negative lens, *f*′_1_ represents the focal length of the glass lens, *f*′_0_ is the focal length of the lens system, *d* is the distance between optofluidic lens and glass lens.

**Figure 6:**
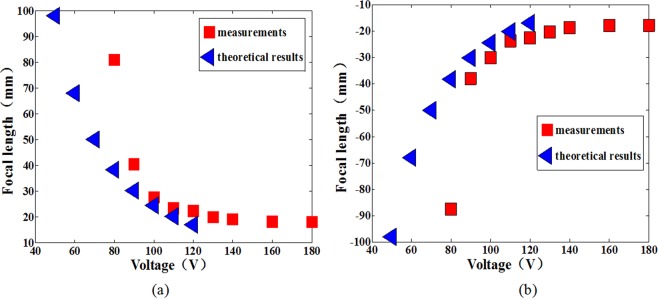
Variation of focal length with voltage. (**a**) Positive focal length changes with the voltage. (**b**) Negative focal length changes with the voltage.

The measured results were presented in Fig. 6. The shortest positive focal length is measured to be ~18.0 mm at ~160 V, and the negative focal length is measured to be ~−17.9 mm at ~160 V. The range of F number varies from 3.6 to infinity. The liquid piston moves up and down in operation. Correspondingly, the focal length changes in both positive and negative directions, which can help enlarge the tuning range of focal length. And it is possible to find a set of Liquid 1 and Liquid 2 with a larger difference of refractive indices to further increase the tuning range of focal length. In addition, there is a big gap between theoretical and measured values of focal length of the proposed lens in the lower voltage region because of the threshold voltage. The theoretical value and the measured value coincide well when the voltage is larger than 90 V as shown in Fig. 6.

In the proposed lens, the two liquids are immiscible and the L-L surface will not be distorted by gravity effect because the two liquid are density-matched. Therefore, the optofluidic lens has good stability. However, compared with solid lens, both refractive indices of liquids are sensitive to temperature. The refractive index of liquid decreases gradually when the temperature rises. From Eq. 3, we can see that the change of refractive indices of two liquids has little effect on the focal length of optofluidic lens. In optical systems, dispersion usually can cause serious chromatic aberrations. Fortunately, the proposed optical fluidic lens has a structure similar to that of a doublet and can eliminate chromatic aberrations in a simpler way by mixing or dissolving selected substances.

## Conclusions

We have demonstrated an optofluidic lens based on electrowetting liquid piston. The proposed lens has two connected chambers, the piston chamber and the lens chamber, to form a closed-loop fluidic system. The electrowetting liquid piston can generate clockwise and counter-clockwise liquid flows, which can make the L-L interface convex and concave. To prove the concept, we fabricate an optofluidic device whose shortest negative and positive focal lengths are ~−17.9 mm and ~18 mm with 5 mm aperture, respectively. The proposed optofluidic lens has large tunable focal length range. Widespread application of such an adaptive lens is foreseeable.

## Supplementary information

Video 1

Video 2
